# Neuroprotective response after photodynamic therapy: Role of vascular endothelial growth factor

**DOI:** 10.1186/1742-2094-8-176

**Published:** 2011-12-16

**Authors:** Misa Suzuki, Yoko Ozawa, Shunsuke Kubota, Manabu Hirasawa, Seiji Miyake, Kousuke Noda, Kazuo Tsubota, Kazuaki Kadonosono, Susumu Ishida

**Affiliations:** 1 Laboratory of Retinal Cell Biology, Keio University School of Medicine, 35 Shinanomachi, Shinjuku-ku, Tokyo 160-8582, Japan; 2 Department of Ophthalmology, Keio University School of Medicine, 35 Shinanomachi, Shinjuku-ku, Tokyo 160-8582, Japan; 3 Department of Ophthalmology, Yokohama City University Medical Center, 4-57 Urafune-cho, Minami-ku, Yokohama, Kanagawa 232-0024, Japan; 4 Department of Ophthalmology, Hokkaido University Graduate School of Medicine, N-15, W-7, Kita-ku, Sapporo 060-8638, Japan

**Keywords:** VEGF, PDT, retina, neuroprotection, Akt, BAX

## Abstract

**Background:**

Anti-vascular endothelial growth factor (VEGF) drugs and/or photodynamic therapy (PDT) constitute current treatments targeting pathological vascular tissues in tumors and age-related macular degeneration. Concern that PDT might induce VEGF and exacerbate the disease has led us to current practice of using anti-VEGF drugs with PDT simultaneously. However, the underlying molecular mechanisms of these therapies are not well understood.

**Methods:**

We assessed VEGF levels after PDT of normal mouse retinal tissue, using a laser duration that did not cause obvious tissue damage. To determine the role of PDT-induced VEGF and its downstream signaling, we intravitreally injected a VEGF inhibitor, VEGFR1 Fc, or a PI3K/Akt inhibitor, LY294002, immediately after PDT. Then, histological and biochemical changes of the retinal tissue were analyzed by immunohistochemistry and immunoblot analyses, respectively.

**Results:**

At both the mRNA and protein levels, VEGF was upregulated immediately and transiently after PDT. VEGF suppression after PDT resulted in apoptotic destruction of the photoreceptor cell layer in only the irradiated area during PDT. Under these conditions, activation of the anti-apoptotic molecule Akt was suppressed in the irradiated area, and levels of the pro-apoptotic protein BAX were increased. Intravitreal injection of a PI3K/Akt inhibitor immediately after PDT increased BAX levels and photoreceptor cell apoptosis.

**Conclusion:**

Cytotoxic stress caused by PDT, at levels that do not cause overt tissue damage, induces VEGF and activates Akt to rescue the neural tissue, suppressing BAX. Thus, the immediate and transient induction of VEGF after PDT is neuroprotective.

## Background

Vascular endothelial growth factor (VEGF) was first identified as a soluble factor that promotes tumor neovascularization [1]. Targeting VEGF has been a key therapeutic strategy for inducing tumor regression [2]. This technology has been widely applied in other fields as well, including treatment of age-related macular degeneration (AMD) [3-5]. AMD is a vision-threatening disease caused by choroidal neovascularization that can secondarily cause irreversible damage to the neural retina. The rationale for targeting VEGF in such diseases is its potential role as a pathogenic factor that promotes deleterious growth of vascular tissues [6-10]. However, VEGF is also a physiological factor [11], indispensable for the maintenance of healthy vessels [12,13] and neurons [14,15]. Since VEGF functions as a double-edged sword, caution is required in its therapeutic use, to make sure that its effect on diseased tissue is desirable. Thus, the physiological roles of VEGF in normal tissue and disease need to be well understood.

Another therapeutic strategy for vascular suppression is photodynamic therapy (PDT) [16,17], which involves the intravenous injection of a photosensitizer, verteporfin, that accumulates in neovascular tissue, which is then irradiated by a low-power laser. Although the degree of laser irradiation is far too low to cause thermal injury, the activated verteporfin generates reactive oxygen species, which are cytotoxic and induce transient thrombosis leading to vessel closure [18]. PDT has been used in anti-tumor therapy to induce regression of feeder vessels [19], and it is now also being used as a treatment for AMD [16,20,21].

A recent study, performed in patients with untreatable ocular malignancy requiring enucleation, showed induction of VEGF after PDT [22]. This isolated study prompted concern that VEGF elevation after PDT could activate growth of residual neovascular tissue. Therefore, these two types of vascular suppressive therapies are sometimes used simultaneously as a combined therapy, in hopes of obtaining greater vascular regression and a better visual prognosis [23]. However, the mechanism of VEGF induction after PDT and its function under these conditions have not been investigated.

The reason for VEGF's induction after PDT could be hypoxia due to normal vessel closure [22], since hypoxia can induce VEGF via DNA binding of hypoxia-inducible factors (HIFs) [24]. However, the stress-response element in the *vegf *gene [25] may be activated by PDT-induced oxidative stress, not only in choroidal neovascularization (CNV) but also in surrounding tissues that receive low-level laser irradiation during PDT. If VEGF is upregulated in response to PDT-induced stress, it may be an important component of the stress-activated biological defense system [26]. In this case, anti-VEGF therapy concomitant with PDT could harm surrounding retinal tissue, which directly affects visual function. Therefore, we decided to investigate the expression response and role of VEGF in the retina after PDT.

In this study, we performed PDT on normal, intact mouse retina, using a laser level below the damage threshold for normal tissue, and analyzed VEGF expression. We also studied the histological consequences of suppressing VEGF function after PDT, and examined the activation of a downstream component of the VEGF signal, Akt, and BAX, a mitochondria-related proapoptotic molecule inhibited by Akt. The use of normal retina in this study, instead of an artificial CNV model induced by high-level laser irradiation, allowed us to simplify the analyses of the biological defense system in the normal retina, and also to study histological changes, since in the CNV model, neural retina has already been damaged during induction of the model, thus PDT-induced damage would be difficult to identify.

## Methods

### Animals and Photodynamic Therapy

All animal experiments described in this study were conducted in accordance with the ARVO (Association for Research in Vision and Ophthalmology) Statement for the Use of Animals in Ophthalmic and Vision Research.

Six-week-old C57BL/6 mice (Clea, Tokyo, Japan) were anesthetized with pentobarbital sodium (70 mg/kg body weight) and immobilized on a stereotactic frame. The pupils were dilated with a mixed solution of 0.5% tropicamide and 0.5% phenylephrine (Mydrin-P^®^; Santen, Osaka, Japan). Verteporfin (3.0 mg/m^2 ^body surface area; Visudyne^®^; Novartis, Basel, Switzerland) was injected into the tail vein as a bolus in a volume of 0.2 ml. Fifteen minutes after the injection, 690-nm laser light was administered using a diode laser (Visulas 690s; Carl Zeiss Meditec, Jena, Germany) delivered through a slit lamp adaptor. The laser spot size was set at 800 μm, and the exposure of the intact retina was 300 μm away from the optic disc, as confirmed by a micrometer. The laser power was set at 600 mW/cm^2^, and it was delivered for 42, 20, or 10 seconds, to yield a fluence of 25, 12, or 6 J/cm^2^, respectively.

### Intravitreous injection of a VEGFR1 Fc fusion protein or LY294002

Animals received 1-μl intravitreous injections of a VEGFR1 Fc fusion protein or LY294002 via an UltraMicro-Pump (type UMP2) equipped with a MicroSyringe Pump Controller (World Precision Instruments, Sarasota, FL) [27], immediately after PDT. A mouse VEGFR1 Fc chimera (R&D Systems) [11] was dissolved in sterile PBS at 0.5, 1, and 2 μg/μl. This fusion protein blocks all VEGF isoforms. LY294002 was dissolved in DMSO at 5 mg/ml and diluted to 10 μM in PBS. For controls, vehicle, either sterile PBS or PBS with the corresponding concentration of DMSO, was injected.

### Histological analysis

Sections were prepared using a protocol described elsewhere [28]. Briefly, retinal samples were fixed with 4% paraformaldehyde and prepared for cryosectioning. Cryosections (9 μm), passing through the optic nerve and the middle of the PDT spot, were prepared. Sections obtained from eyes 7 days after PDT were stained with hematoxylin and eosin, and those obtained 3 days after PDT were used for TUNEL assays and immunostaining. TUNEL staining was performed according to the manufacturer's protocol (ApopTag Fluorescein In Situ Apoptosis Detection Kit; Chemicon, Temecula, CA) and as previously described [29]. TUNEL-positive cells were counted and the average number per section was calculated. To detect pAkt, endogenous peroxidase was abolished by incubating sections in 3% (wt/vol.) hydrogen peroxide in methanol for 20 min. Sections were then incubated in blocking solution (10% normal bovine serum in PBS), and then with a rabbit anti-pAkt antibody (1:25; Cell Signaling Technology), followed by a biotinylated secondary antibody and avidin-biotin horseradish peroxidase complexes (Vectastain Elite ABC Kit). The reaction product was developed by incubation for 10 min in Tyramide Signal Amplification Solution (Perkin Elmer Life Sciences, Boston, MA, USA). Nuclei were counter-stained with bisbenzimide at a 1:1000 dilution of a 10 mg/mL stock solution (Hoechst 33258; Sigma). All the sections were examined using a microscope equipped with a digital camera (Carl Zeiss, Jena, Germany).

### Real-time (RT)-PCR

Total RNA was isolated from the retina with TRIzol (Invitrogen, Carlsbad, CA) and reverse-transcribed with a cDNA synthesis kit (First-Strand; Amersham Biosciences, Inc., Piscataway, NJ), according to the manufacturers' protocols. PCR was performed with TaqMan^® ^Fast Universal PCR Master Mix in an Applied Biosystems 7500 Fast real time PCR system (Applied Biosystems, Foster City, CA). The primers were the TaqMan probes for β-*actin *and *vegf *A. The results are presented as the ratio of the mRNA of *vegf *to that of an internal control gene, β-*actin*.

### ELISA

The neural retina or retinal pigment epithelium (RPE)-choroid complex of each mouse was carefully isolated and placed into 100 μl of lysis buffer (0.02 M HEPES, 10% glycerol, 10 mM Na4P2O7, 100 μM Na3VO4, 1% Triton, 100 mM NaF, 4 mM EDTA [pH 8.0]) supplemented with protease inhibitors [30]. After sonication, the lysate was centrifuged at 15,000 rpm for 15 minutes at 4°C. The protein level of VEGF in the supernatant was determined with a mouse VEGF ELISA kit (R&D Systems, Minneapolis, MN), according to the manufacturer's instructions. The tissue concentration was calculated from a standard curve and corrected for protein concentration as evaluated by the NanoDrop ND-1000 spectrophotometer (Thermo Fisher Scientific, Waltham, MA), as previously described [28].

### Immunoblot analyses

Isolated retinas were placed into lysis buffer (10 mmol/l TRIS-HCl [pH 7.6], 100 mmol/l NaCl, 1 mmol/l EDTA, 1% [wt/vol] Triton X-100, and protease inhibitors) as previously described [31]. Each sample was separated by SDS-PAGE and electroblotted onto a polyvinylidene fluoride membrane (Millipore, Bedford, MA, USA). After being blocked in TNB buffer, the membrane was incubated at 4°C overnight with a rabbit polyclonal anti-phospho-Akt antibody (1:1,000; Cell Signaling), anti-BAX antibody (1:1,000; Cell Signaling), and mouse monoclonal anti-α-tubulin antibody (1:10,000; Sigma-Aldrich), respectively. The signals were visualized by chemiluminescence (ECL Blotting Analysis System; Amersham, Arlington Heights, IL, USA), measured by Image J software, and normalized to α-tubulin.

### Statistical analyses

All results are expressed as mean ± SD. The values were assessed for statistical significance (Mann-Whitney test), and differences were considered significant at *P *< 0.05.

## Results

### Defining the conditions for PDT in mice

We first evaluated histological changes in the mouse retina after PDT of various durations, to define appropriate sub-damage threshold irradiation period for our analysis. We injected verteporfin at 3 mg/m2, and performed low-level laser treatments 300 μm away from the optic disc for 42, 20, or 10 seconds.

**Figure 1:**
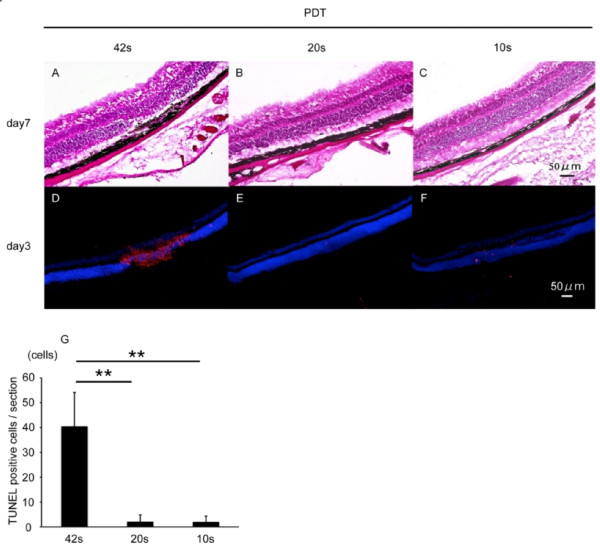
**Defining PDT duration for mice**. (A-C) Hematoxylin-eosin staining of a retinal section 7 days after PDT. The photoreceptor cell layer was thin in the area irradiated for 42 seconds using low-level laser light during PDT (A). No obvious changes were observed in retinal sections exposed to 20 or 10 seconds of laser irradiation (B,C). (D-G) TUNEL (red) and Hoechst (blue) stainings of retinal sections 3 days after PDT. TUNEL-positive cells were observed in the photoreceptor cell layer in only the irradiated area of retinas exposed to 42 seconds of laser light (D). Few TUNEL-positive cells were observed in areas exposed to 20 or 10 seconds of laser irradiation (E,F). TUNEL-positive cells in retinal sections were counted (G). Scale bar, 50 μm. **p < 0.01.

In sections of retina obtained 7 days after PDT, the photoreceptor cell layer was thinned at the site of irradiation and showed a loss of photoreceptor cells at the longest (42-second) PDT duration (Figure 1A). No obvious thinning was seen in retinal sections treated with PDT for 20 or 10 seconds (Figure 1B,C). We next performed TUNEL assays in sections of retina obtained 3 days after PDT. In sections irradiated for 42 seconds, obvious TUNEL-positive labeling was observed in the photoreceptor cell layer (Figure 1D,G). Almost no positive cells were observed after 20 or 10 seconds of PDT (Figure 1E-G). The changes after PDT were observed only in the irradiated area. The remainder of the retina was intact; thus the significance of retinal histological changes in the irradiated area were well defined by comparison with the non-irradiated retina.

On the basis of these preliminary findings, we performed PDT for 20 seconds in the following experiments, since this duration of PDT did not cause obvious morphological changes in the neural retina.

### VEGF induction in the retina after PDT

**Figure 2:**
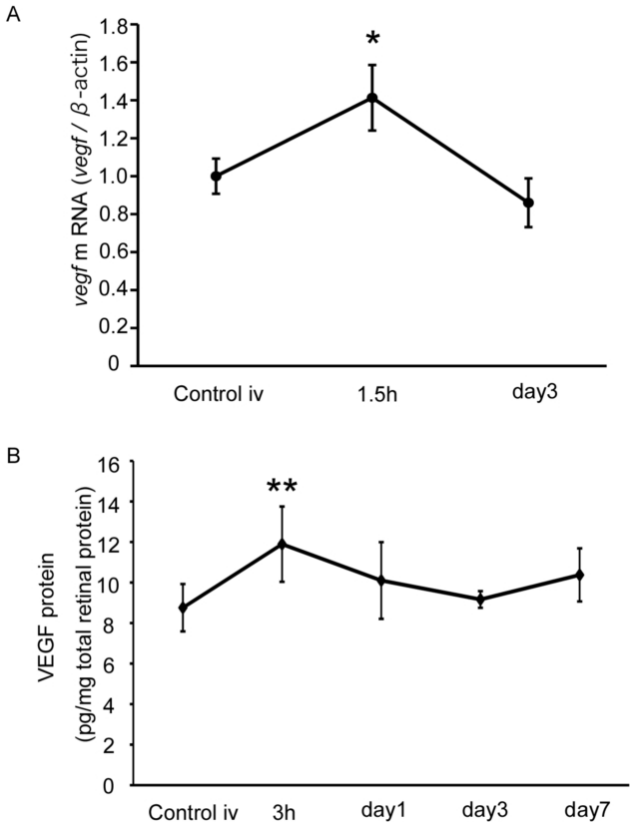
**VEGF induction in retina after PDT**. (A) *vegf *mRNA expression in neural retina analyzed by real-time (RT)-PCR. *vegf *mRNA was upregulated transiently 1.5 hours after PDT. (B) VEGF protein expression analyzed by ELISA. VEGF protein increased transiently 3 hours after PDT. *p < 0.05, **p < 0.01.

Next, we analyzed VEGF levels after PDT. mRNA levels measured by real time (RT)-PCR showed a peak increase 1.5 hours after PDT that returned to baseline by 3 days after PDT (Figure 2A). By ELISA, VEGF protein levels peaked 3 hours after PDT, and also gradually decreased and returned to baseline by 3 days post-PDT (Figure 2B). Thus, both VEGF mRNA and protein levels increased immediately and transiently in the retina after PDT.

### Influence of VEGF inhibition after PDT

**Figure 3:**
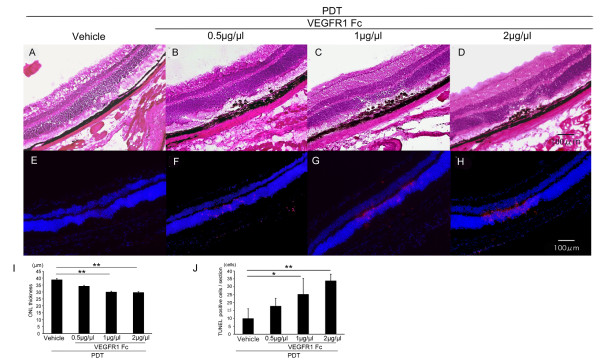
**Influence of VEGF inhibition after PDT**. (A-D) Hematoxylin-eosin staining of a retinal section 7 days after PDT with VEGF inhibition. Damage to the photoreceptor cell layer was obvious when VEGFR1 Fc was injected into the eye immediately after PDT (B-D). (E-H) TUNEL (red) and Hoechst (blue) stainings of retinal sections 3 days after PDT with VEGF inhibition. TUNEL-positive cells increased when VEGFR1 Fc was injected after PDT (F-H). The thickness of photoreceptor cell layer in the center of the lesion was measured (I) and the number of TUNEL-positive cells were counted (J). The effects were dose-dependent. Scale bar, 100 μm. *p < 0.05, **p < 0.01.

To explore the role of increased VEGF after PDT, we injected a VEGF inhibitor, VEGF receptor 1 Fc chimera (VEGFR1 Fc) immediately after PDT and analyzed retinal sections 7 days later. Sections of control retinas, treated with vehicle and PDT, showed no histological changes (Figure 3A). However, in sections from PDT and VEGFR1 Fc-treated retinas, the thickness of the photoreceptor cell layer was reduced in the irradiated area, with a dose-dependent increase in severity from 0.5 μg/μl to 2 μg/μl (Figure 3B-D,I). We also found a dose-dependent increase in the number of TUNEL-positive cells in the ONL, 3 days after the combined PDT and VEGFR1 Fc treatment (Figure 3E-H,J). These data show that VEGF inhibition immediately after PDT promotes photoreceptor cell apoptosis.

### Influence of VEGF inhibition on Akt and BAX

**Figure 4:**
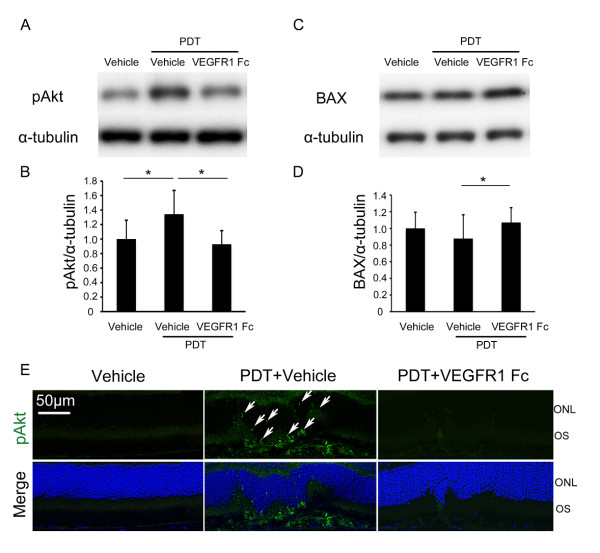
**Influence of VEGF inhibition on Akt and BAX**. (A-D) Immunoblot analyses. One day after PDT with vehicle injection, pAkt levels increased, but levels were decreased by VEGF inhibition with injection of VEGFR1 Fc (2 μg/μl) into the eye immediately after PDT (A,B). BAX levels in the retina were not changed by PDT when vehicle was injected, but increased when VEGFR1 Fc (2 μg/μl) was injected immediately after PDT (C,D). (E) Immunohistochemistry. Three days after PDT, pAkt was observed in photoreceptor cells, in cell bodies and outer segments (arrows), of the irradiated area of the vehicle-treated retina, but little staining was observed in the VEGFR1 Fc (2 μg/μl)-treated retina. pAkt, phosphorylated Akt, ONL, outer nuclear layer, OS, outer segment. *p < 0.05, **p < 0.01.

We next analyzed the influence of VEGF inhibition on Akt activation in PDT-treated retina. Immunoblot analysis showed that, one day after PDT, levels of phosphorylated (i.e., activated) Akt increased in PDT-treated retina compared with retina with no PDT, under the vehicle injection condition. But with VEGF inhibition by injecting VEGFR1 Fc (2 μg/μl) immediately after PDT, levels were significantly lower than with vehicle injection after PDT (Figure 4A,B). In contrast, levels of BAX were significantly higher when VEGF inhibition was combined with PDT, although the level was not changed by PDT under the vehicle injection condition (Figure 4C,D). In retinal sections 3 days after PDT, phosphorylated Akt appeared in the photoreceptor cells of the irradiated area, but such staining was hardly observed in VEGFR1 Fc (2 μg/μl)-treated retina (Figure 4E).

### Influence of Akt inhibition after PDT

**Figure 5:**
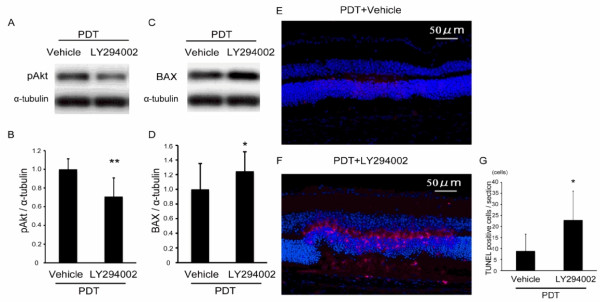
**Influence of Akt inhibition after PDT**. (A-D) Immunoblot analyses. pAkt levels decreased (A,B) and BAX levels increased (C,D) in retina 1 day after a PI3K inhibitor (LY294002, 10 μM) was injected into the eye. (E-G) TUNEL (red) and Hoechst (blue) stainings 3 days after PDT. LY294002 injection increased the number of TUNEL-positive cells in the irradiated area. pAkt, phosphorylated Akt. Scale bar, 50 μm. *p < 0.05.

We next investigated whether reduced Akt signaling was involved in apoptosis of photoreceptor cells after PDT. To do this, we inhibited an upstream component of the Akt signal, PI3K, by injecting LY294002 (10 μM) into the retina immediately after PDT. First, we confirmed that the injection suppressed levels of phosphorylated and activated Akt 1 day after PDT (Figure 5A,B), and found that BAX levels increased in the same retina (Figure 5C,D). Three days after the treatments, TUNEL assay labeled cells in the laser-irradiated area only when LY294002 was injected after PDT, in contrast to vehicle injection with PDT (Figure 5E-G). Therefore, Akt activation after PDT promoted retinal cell survival.

## Discussion

Here we demonstrate that the VEGF expression that is induced in mouse retina after PDT is neuroprotective. VEGF inhibition suppressed Akt activation and increased BAX levels in retina, leading to photoreceptor cell apoptosis after PDT, but only within the irradiated area. Suppression of Akt activation with a PI3K inhibitor also increased BAX expression and apoptosis of irradiated photoreceptor cells. Thus, VEGF plays a neuroprotective role, activating Akt, in the stressed retina.

Since inhibition of VEGF induced apoptosis of photoreceptor cells in the irradiated area, PDT caused pro-apoptotic stress. This stress was compensated for by VEGF expression in retinas treated with PDT alone. This finding is consistent with VEGF's reported role in other neural systems. For example, brain infarction induces VEGF expression, and administration of VEGF reduces brain damage after stroke [15,26,32,33]. Alternatively, transgenic mice expressing reduced levels of VEGF because of a mutant VEGF promoter show neurodegeneration similar to amyotrophic lateral sclerosis (ALS); this neurodegeneration is prevented by VEGF treatment [34]. In another knock-in mouse model, in which the hypoxia response element sequence in the vegf promoter is deleted, reduced VEGF expression leads to motor neuron degeneration [14].

**Figure 6:**
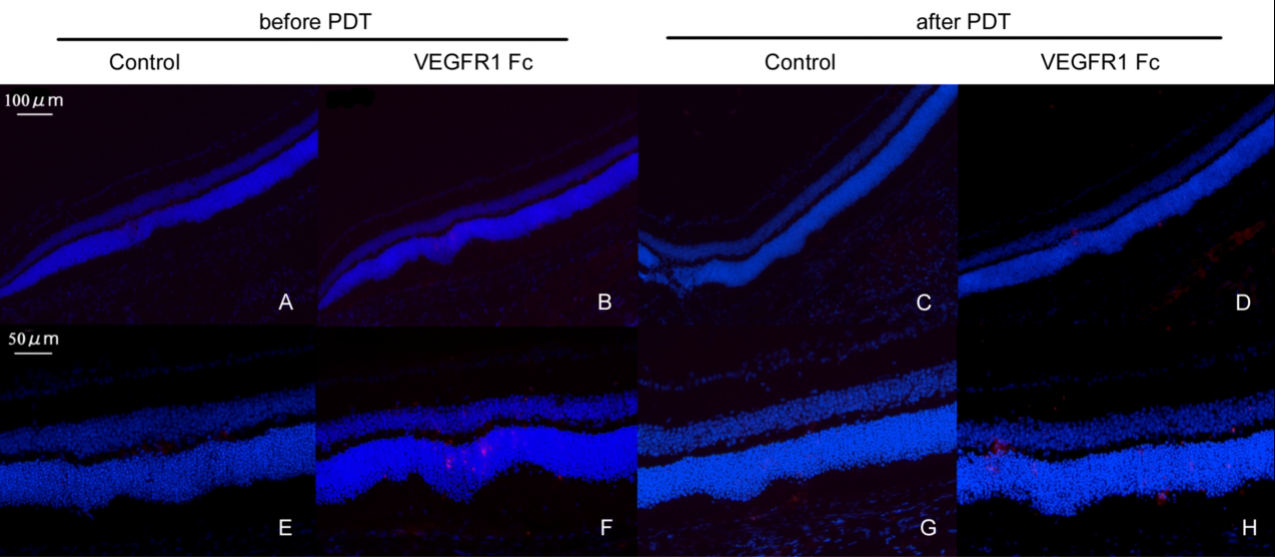
**TUNEL staining after PDT and VEGF inhibition**. (A-H) TUNEL (red) and Hoechst (blue) stainings of retina. Eyes were injected with VEGFR1 Fc (2 μg/μl) 7 days before PDT, and retinas were fixed 3 days after PDT (A,B,E,F). Or, VEGFR1 Fc (2 μg/μl) was injected 7 days after PDT, and retinas were fixed 3 days after injection (C,D,G,H). Almost no TUNEL staining is observed in the retina. Scale bar, 100 μm (A-D), 50 μm (E-H).

The influences of inhibiting VEGF expression after PDT were not observed outside the irradiated area, which indicates that this level of VEGF inhibition is not cytotoxic for non-irradiated and non-stressed retinal cells. Moreover, the induction of VEGF in the irradiated retina was immediate and transient, suggesting that VEGF's function under these conditions may be required in a specific time window after stress. We obtained support for this idea by inhibiting VEGF in retina 7 days before or after PDT, and finding almost no apoptotic cells in the irradiated area (Figure 6). Therefore, VEGF inhibitor injected 7 days before PDT may not persist and inhibit VEGF's action in the retina immediately after PDT. On the other hand, the retina may have recovered from the stress within 7 days from PDT, as we found that VEGF inhibition 7 days after PDT at this level did not cause photoreceptor cell death.

In this study, both VEGF and Akt promoted survival of irradiated photoreceptor neuronal cells, reducing BAX, an apoptotic Bcl-2 family protein. VEGF's activation of Akt is consistent with a previous report showing that Akt activation occurs downstream of VEGF receptor (VEGFR) 2 signaling [35]. There are reports that VEGFR 2 signaling [36] and Akt [37] both inhibit the activation and translocation of BAX to mitochondria. What we observed, however, was a decrease in BAX protein levels when the VEGF-Akt pathway was activated. Recent papers report that Akt phosphorylates BAX, which shortens its half-life as well as blocking its translocation [38,39]. A reduced half-life of BAX may be, at least in part, the mechanism responsible for the biological defense system induced by PDT. The finding in this study that phosphorylated Akt is expressed in the cytoplasm of photoreceptor cells (Figure 4E arrows) supports this idea.

Cytological changes after PDT have been shown in a crayfish stretch receptor that consists of a single sensory neuron enwrapped by glial cells. These include swelling of some mitochondria, the Golgi apparatus, and endoplasmic reticulum cisterns [40]. The changes in the mitochondria and Golgi apparatus are the first to occur and persist the longest, and therefore these subcellular organelles are judged to have the greatest sensitivity to PDT [41]. Our finding that BAX, an essential molecule for the mitochondrial apoptotic pathway, increased after PDT when VEGF was inhibited is consistent with the histological findings in the crayfish.

PDT is a widespread treatment for AMD, as is anti-VEGF therapy. The latter is a leading treatment for AMD, but requires repeated treatments and a significant investment of time on the part of patient and doctor. In contrast, PDT has a rapid effect. Thus, a recent trial was undertaken to combine PDT and anti-VEGF therapy, in order to shut down CNV as quickly as possible. In addition, the possibility that VEGF might be elevated after PDT-mediated vascular occlusion because of the resulting hypoxia [22] has provided a popular rationale for such simultaneous combined therapy.

**Figure 7:**
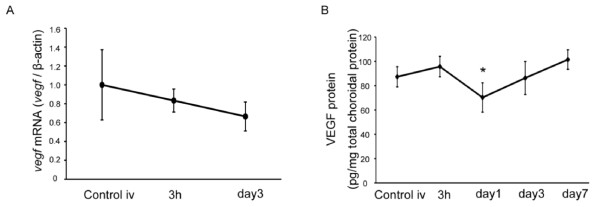
**VEGF expression in the RPE-choroid complex**. (A) *vegf *mRNA expression in the RPE-choroid complex analyzed by real-time (RT)-PCR. *vegf *mRNA did not increase after PDT. (B) VEGF protein expression analyzed by ELISA. VEGF protein decreased transiently 1 day after PDT. *p < 0.05.

Here, we found that VEGF levels after PDT increased only transiently in neural retina. Moreover, VEGF is physiologically secreted by RPE and most probably by choroidal components, but is not induced and rather reduced in RPE-choroid complex after PDT (Figure 7). Because the VEGF inhibition immediately after PDT also induced apoptosis of RPE as observed in Figure 3, RPE would be affected by PDT and would required VEGF's action to avoid apoptosis. Thus, the production of VEGF might be influenced by a decrease in bioactivity of RPE cells. This is consistent with previous data showing a trend toward reduced density of the choriocapillaris, which is positively regulated by VEGF under physiological condition, in the irradiated areas of retina when treated in combination with an anti-VEGF drug in monkey eyes [42]. Although our current study did not examine the effects of VEGF blockade on PDT-treated CNV, overall VEGF levels after PDT may not be elevated as highly as assumed. Our results suggest that the use of an anti-VEGF drug simultaneously with PDT might not be a suitable treatment to protect visual function considering the side effects. Combined therapy may promote photoreceptor cell death and visual function impairment, based on our results. Instead, allowing the tissue to regulate its VEGF level may be more important for optimizing neuroprotection and retinal function. Alternatively, adjuvant therapy to increase Akt activation may be beneficial in clinical applications. In any case, the molecular mechanisms underlying any treatment should be considered when establishing a protocol for combination therapy. Given that PDT can be used to treat solid organ tumors, such as cancer of the lung or brain [43], and may cause side effects by damaging intact tissue, we hope our data will help improve patient prognosis after PDT treatment in the fields of oncology as well as ophthalmology.

## Conclusions

The immediate and transient induction of VEGF in response to PDT is neuroprotective and is required for photoreceptor cell survival, activating Akt which inhibits BAX. Since VEGF functions as a double-edged sword, an understanding of its roles in each context is required to establish better therapeutic protocols leading to better prognosis.

## List of abbreviations

VEGF: vascular endothelial growth factor; VEGFR1: vascular endothelial growth factor receptor 1; PI3K: Phosphoinositide 3-kinase; AMD: age-related macular degeneration; PDT: photodynamic therapy; TUNEL: terminal deoxynucleotidyl transferase-mediated dTTP nick-end labeling

## Competing interests

The authors receive financial support from NOVARTIS Pharmacetutical Co., Ltd.

## Authors' contributions

All the authors have read and approved the final version of the manuscript.
